# Design and Prototype Fabrication of a Cost-Effective Microneedle Drug Delivery Apparatus Using Fused Filament Fabrication, Liquid Crystal Display and Semi-Solid Extrusion 3D Printing Technologies

**DOI:** 10.3390/mi13081319

**Published:** 2022-08-15

**Authors:** Petros Papadimitriou, Eleftherios G. Andriotis, Dimitrios Fatouros, Dimitrios Tzetzis

**Affiliations:** 1 Digital Manufacturing and Materials Characterization Laboratory, School of Science and Technology, International Hellenic University, GR-57001 Thessaloniki, Greece; 2 Laboratory of Pharmaceutical Technology, Department of Pharmaceutical Sciences, Aristotle University of Thessaloniki, GR-54124 Thessaloniki, Greece

**Keywords:** drug delivery, hollow microneedles, 3D printing, FFF, LCD, semi-solid extrusion

## Abstract

The current study describes the design of a cost-effective drug delivery apparatus that can be manufactured, assembled, and utilized as easily and quickly as possible, minimizing the time and expense of the supply chain. This apparatus could become a realistic alternative method of providing a vaccine or drug in harsh circumstances, including humanitarian disasters or a lack of medical and nursing staff, conditions that are frequently observed in developing countries. Simultaneously, with the use of microneedles (MNs), the apparatus can benefit from the numerous advantages offered by them during administration. The hollow microneedles in particular are internally perforated and are capable of delivering the active substance to the skin. The apparatus was designed with appropriate details in computer aided design software, and various 3D printing technologies were utilized in order to fabricate the prototype. The parts that required minimum accuracy, such as the main body of the apparatus, were fabricated with fused filament fabrication. The internal parts and the hollow microneedles were fabricated with liquid crystal display, and the substance for the drug loading carrier, which was an alginate gel cylinder, was fabricated with semi-solid extrusion 3D printing.

## 1. Introduction

Drug administration via microneedles (MNs) offers several benefits over the classical routes of administration (e.g., oral administration), such as elimination of pain, avoidance of first hepatic effect, acceptance from people suffering from needle phobia, etc. [1,2]. In addition, transdermal administration can prevent the discomfort, injuries, and infections associated with needle use, whether because of medical staff’s lack of understanding or as a result of the individuality of each human body. Most importantly, microneedles can be utilized on patients who are needle-phobic or have difficulties adapting [3,4,5,6,7,8,9,10].

The administration of actives through the skin using microneedles has many practical approaches, e.g., for antibiotic administration in case of infections, delivery of insulin to patients with diabetes mellitus, heparin as thromboprophylaxis, or morphine to patients with chronic pain or who are severely ill [3].

There are several kinds of different devices and mechanisms using several mechanical principles and types of microneedles, including, among others, solid and hollow microneedles [4]. Microneedles offer considerable comparative benefits over conventional methods for medication and vaccine delivery through the skin.

An appealing feature of microneedles is their sustainability [5]. Over 16 billion injections are performed annually worldwide, a figure that is constantly increasing, particularly during the COVID-19 era. However, when a needle is used, massive amounts of non-biodegradable waste are generated, which have a severe effect on the environment. Thus, microneedles, when combined with appropriate medical devices, can be an effective tool for decreasing medical waste on a global scale [1,8,11]. Despite the numerous advantages, one of the primary drawbacks of intradermal delivery injections with a microneedle is insufficient injection due to leakage. Complete injection was defined as when leakage (non-injected fluid residue on the skin) was less than 10% of the amount of the product to be injected [6,12,13]. As a result, there are precise requirements that medical equipment must meet in order to perform the procedure accurately and effectively.

Additive manufacturing (AM) or 3D printing can potentially play a key role in this process. Three-dimensional printing’s precise spatial control delivers great resolution and precision [14,15,16]. It is widely used in biomedical applications and provides medical solutions, such as the transplantation of human tissues or organs for regenerative medicine [17]. It is also found in medication delivery devices, including printed polypills, microneedles, and customizable devices [18,19,20,21]. Three-dimensional printing is a viable alternative to conventional manufacturing processes because it enables the production of highly configurable and sophisticated microneedle designs without the large capital investments often associated with other industrial-scale approaches [22,23]. The printer constructs the model by layering appropriate materials (e.g., ceramics, liquids, resins, plastic, photopolymer, and powders). Microneedle (MN) arrays were successfully manufactured using additive manufacturing methods like as stereolithography (SLA), liquid crystal display (LCD), and two-photon polymerization (2PP). These cutting-edge technologies offer numerous advantages over traditional manufacturing methods, including simplicity, low cost, the ability to fabricate complex geometrical products with the ability to modify the original designs at any time, and the ability to manufacture patient-specific devices [20,24,25,26,27]. Three-dimensional printing enables the design of desired MN geometries using computer-aided design (CAD) software and their materialization via 3D printing equipment. The ability to store and modify designs provides ease and versatility. This, together with the ease with which such a technique may be implemented, has sparked scientific interest in MN creation via additive manufacturing technologies [23,25].

In recent years, an interactive discussion has been initiated by various healthcare stakeholders about the development of personalized medications at the point of care. Personalized medicine can be described as medicine tailored to suit individual patients’ treatment, including the dose adjustment, size/shape, appearance, taste, texture, smell, ingredient composition, and drug dissolution rate. Advances in additive manufacturing technologies render 3D printing an ideal and novel manufacturing approach for “smart” design and fabrication of personalized dosage forms. Three-dimensional printing of medicine at the point of care will not only address the above issues but will also offer decentralized manufacturing with shorter production times combined with dose flexibility that meet the demands of patients with different clinical needs simultaneously. 

The aim of this present work is the study, design, and construction of a microneedle apparatus that will be able to be used in harsh circumstances all over the world and that can be constructed, assembled, and used by any person without the need for any specific knowledge. In addition, such a device could be used to administer home remedies to the elderly or people with mobility problems who need regular medication. Considering the non-hazardous operation of the device, it could become a permanent solution to administer a predetermined dose of the drug with regard to its pharmacokinetics. This specific apparatus must satisfy some special demands, such as the control of the volume of the liquid substance that passes through hollow microneedles, while using an absorbable gel for drug release. Certain designs are presented along with a final prototype with all specific details and drawings as well as a prototype fabrication.

## 2. Device Development

### 2.1. Goals of the Design

One of the design goals for the proposed device is having it consist of components that are simple to manufacture and assemble. If it is to be utilized in harsh situations, such as humanitarian disasters or a lack of medical or nursing staff, which is frequent in underdeveloped countries around the world, it must be able to withstand those conditions. A major advantage of 3D printing is that it is a tested and effective process that can be utilized and transferred anywhere in the world due to the small size of a 3D printer, which makes it easy to carry. As a result, the manufacture of components becomes simpler when compared to traditional processing methods, such as mold machining and injection molding, which require huge and non-portable installations, as well as highly trained employees for their use. In addition to the way the parts are constructed, the device must have a relatively basic “principle of operation” that is simple to comprehend and does not necessitate the supervision of professional employees during the assembly process. As a result, methods and components were chosen, such as designed plastic parts, but also standardized screws, that did not necessitate the use of specialist tools or knowledge, allowing for the assembly and disassembly of the mechanism to be straightforward. The simplicity of the parts definitely has an impact on the cost, which is clearly decreased when only a few different parts exist. 

Along with the disassembly phase, the replacement of previously used materials is critical. These elements include the gel containing the active drug substance, as well as the microneedle array, which must be replaced after each usage, as it cannot be used from patient to patient in order to avoid disease transmission. The gel with the active drug should be able to be removed with fingers or another straightforward tool and then replaced with a new one. The gel that will contain the drug can be also made by 3D printing methods. To accomplish correct deposition, it is vital to select gel materials with sufficient connectivity mechanisms and rheological qualities. Thus, the material must be viscous enough to be released but also rigid and strong enough to retain its structural integrity after 3D printing. Additionally, it must display appropriate swelling characteristics and short-term stability, so that its original mechanical qualities are retained, and no collapse occurs during final construction [12]. Three-dimensional printing enables creation in a variety of geometries and shapes [13]. Such freedom broadens the range of feasible configurations for the device’s design, size, and operating principle.

A distinguishing feature that it is desirable for the apparatus to have is detailed control over the supply of the liquid substance. In some cases, active substances require extremely sophisticated administration in terms of the volume provided. Another critical consideration is the device’s size and weight. Initially, the device should be compatible with the average adult patient, but should also potentially be usable in children. The weight should be light enough to ensure comfort during use. This is critical, as drug distribution via microneedles is not instantaneous and requires some time for completion. Therefore, the device must, during use, be tightly connected to the patient’s hand, so that the active substance does not leak from the microneedles.

The presence of microneedles is a great advantage over a conventional needle, as they do not create a feeling of fear in the patient and especially in those suffering from needle phobia [7]. In general, the shape and form of the device should not be repulsive and should not cultivate the feeling of fear in the patient. On the contrary, the diligent design should make the device attractive to the patient and gives him a sense of security. As previously stated, the apparatus is not intended for use by specialized individuals and must be capable of being constructed/assembled by anyone. Apart from the assembly, however, its practical application must be able to administered by the user or an inexperienced individual, not necessarily by a doctor or nurse. The objective is to include these features in the design and implementation of the device, thereby making it perfect for use in any location and under any conditions.

### 2.2. Design Proposal—Function Analysis

**Figure 1:**
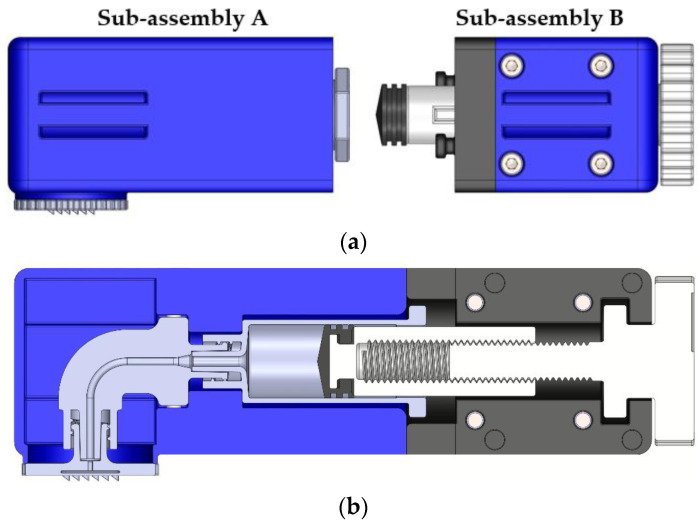
(**a**) Preparation for connection of A and B sub-assemblies; (**b**) sectional view of the drug delivery apparatus.

The proposed assembly consists of eight parts, in addition to the screws, nuts, and the seal. The overall arrangement could conceivably be divided into two sub-assemblies as shown in Figure 1, one of which starts from the rotation lever (arrangement B) and the other ends in the MN array (arrangement A). The two sub-assemblies are joined together so that the planes of their surfaces coincide.

**Figure 2:**
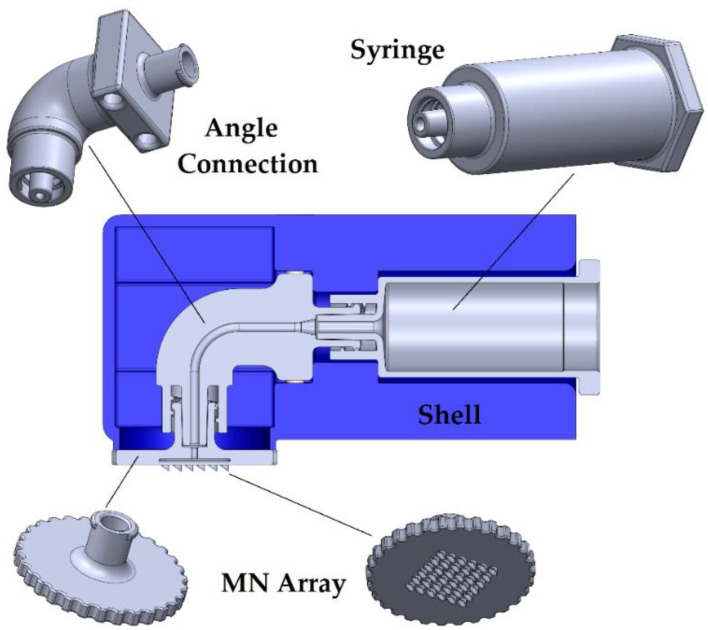
Sectional view of sub-assembly A.

Sub-assembly A consists of four custom-designed parts as illustrated in Figure 2 as well as two standard M3×35 Allen screws and two simple M3 nuts. The part “Shell”, which is visible from the outside of the device, contains special configurations and cavities to connect with it the neighboring parts. Its dimensions are 40 × 40 × 80 mm. Externally, there are slots through which Velcro straps can pass to mount the device on the arm. Inside the “Shell”, a cavity exists to nest the “Angle Connection” corner connection. The “Angle Connection” contains at each end a standard Luer lock thread, so that it can be connected on one side with the syringe and on the other side with the microneedle array (“MN Array”). The lower thread of the angle connector is male Luer lock, and the upper is female. Respectively, the “MN Array” has female threads, whereas the syringe has male threads. The link is also visible along with the internal channel, which has a diameter of 1 mm for the active drug to pass through.

The “Angle Connection” is positioned so that it cannot be rotated and remains fixed radially. In terms of axial mounting, there are two holes in the hexagon of the connector that are concentric and have corresponding holes in the “Shell” part. Once the “Angle Connection” is in place, Allen screws pass through holes in the two parts. There they lock with Allen M3 nuts, for which there are two sockets that do not allow them to rotate.

The “MN Array” can be manually screwed to the “Angle Connection”. The screwing is complete at the same time that its surface comes in contact with the circular surface that protrudes at the bottom of the “Shell”, when the sealing is ensured. In a similar way, the “MN Array” is unscrewed and replaced by the new one purposed for the next usage. The circular pattern with the cuts around the perimeter exists to facilitate the screwing and unscrewing of the part by hand. On the lower surface of the “MN Array”, the microneedles are placed, which are hollow for a continuous flow of the drug substance. The geometry of the microneedles is an elliptical internal channel with a small diameter of 1 mm, and a large diameter of 1.3 mm, whereas the external shape is circular with a diameter of 2 mm. The microneedles should be sharp at the edge, so they are designed with an angle of 36.25°. The array consists of a 6 × 6 arrangement, i.e., a total of 36 microneedles, which are lined up with a distance of 2 mm center to center from each neighboring microneedle. In order for the liquid to pass through the entry of the part into the microneedles, there is first a small channel with a diameter of 1 mm, from which it enters a 0.5 mm high case, and then the microneedles are delivered directly to the subcutaneous tissue of the patient. The connection of the “Angle Connection” to the “Syringe”, which has a male Luer Lock thread, has the same mode of operation.

**Figure 3:**
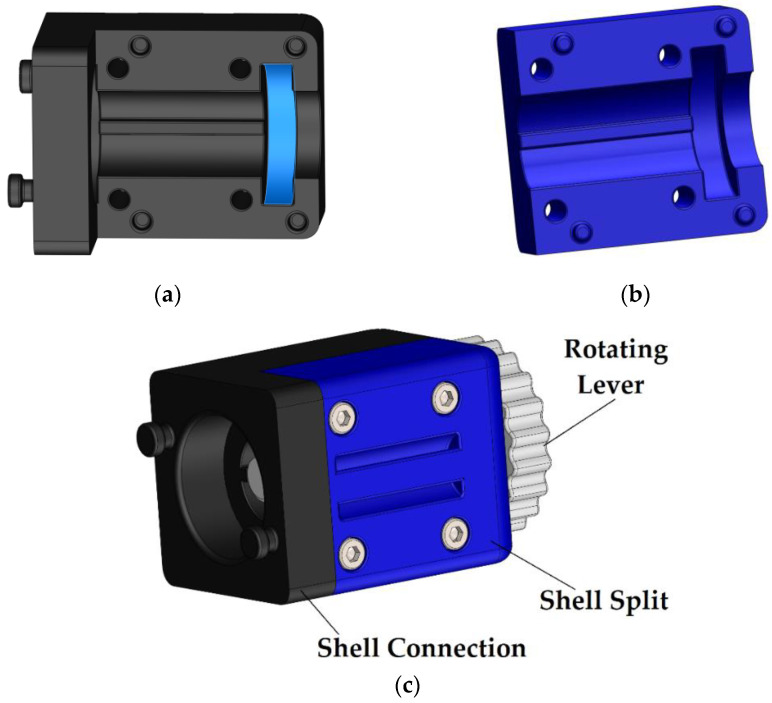
(**a**) General view of “Shell Connection” with marked surfaces for mounting cylinder; (**b**) general view of “Shell Split”; (**c**) view of “Shell Connection”, “Shell Split”, and “Rotating Lever”.

Its internal dimension has a diameter of 15 mm, a standard syringe of 12 mL, a path length of about 35 mm, and a wall thickness of 0.8 mm. Screwing can be done by hand, as it has a hexagon on the back for convenience and the screwing is complete when the surface of the front of “Syringe” is in contact with the configuration of the “Shell” part. Sub-assembly B consists of four designed parts and four M3 Allen screws plus their nuts. The “Shell Connection” connects the two sub-assemblies. To assemble this layout, a “Rotating Lever” is inserted into the unique cylindrical arrangement, followed by the “Shell Split,” which is placed in the “Shell Connection” corresponding holes through its four pins as shown in Figure 3. The pins are necessary to align the exterior surfaces of the A and B sub-assemblies. The connection is then secured using four Allen M3 screws that pass through the “Shell Split” and have their upper surface aligned with the part’s outer surface with grooves. In the “Shell Connection”, hexagonal pockets are provided for the nuts. This allows for the screws to be tightened with a simple Allen wrench. The reason for the two divided sections is that the cylindrical part of the “Rotating Lever” should not move axially and is thus trapped inside the particular case in which it rests. For the optimum fit of the parts and given the construction possibilities and defects, there is an axial grace of about 0.5 mm between the “Connection Shell” and the “Shell Split”, so that they do not rub against the “Rotating Lever” and make rotation difficult.

**Figure 4:**
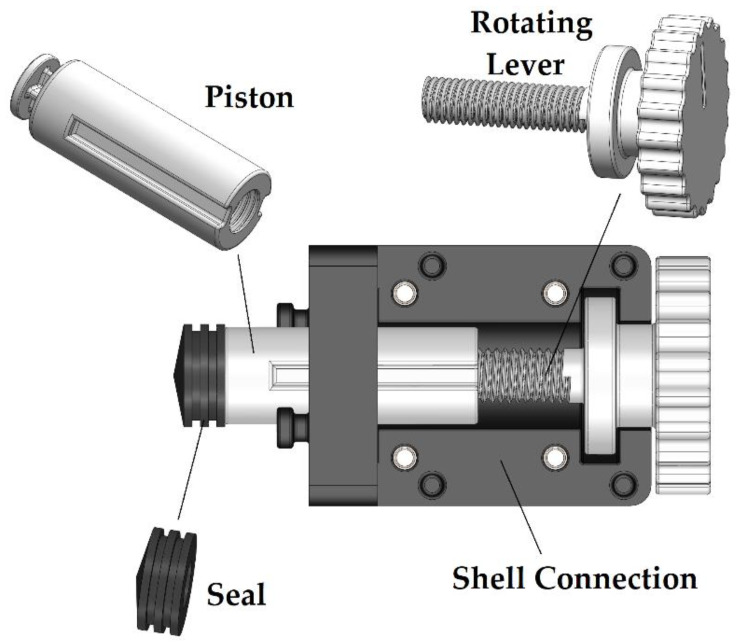
Internal view of sub-assembly B.

To implement this property, a mechanism was selected that converts the rotational motion imposed by the user into a linear one as shown in Figure 4. More specifically, the part that will carry the rubber seal on its front has internally, almost along its entire length, a female thread (“Piston”) that is counterclockwise. In addition, the piston has a special configuration at the front so that it can apply the sealing element, which is standardized by a 12 mL Luer Lock syringe.

The “Rotating Lever” has some digging on the outside to create enough friction for the user to effortlessly rotate it. The cylindrical surface has a larger diameter to prevent the “Rotating Lever” from moving in a straight line. On the front of the “Rotating Lever” is a male thread that connects to the female “Piston” thread. This assembly’s purpose is to convert the rotating motion into a straight motion for the “Piston”. 

To incorporate this mechanism into the proposed design, a female groove has been carved into the cylindrical surface of the “Piston”, as well as a corresponding male configuration in the “Shell Connection” and “Shell Split” components that contain the “Piston”. Such design approach does not allow the “Piston” to rotate, while when it comes in contact with the “Rotating Lever” thread and this rotates then the “Piston” is forced to move linearly. This is the principle of operation of this mechanism. Thus, knowing the pitch of the thread, the axial displacement is known and recorded by the “Piston” when the “Rotating Lever” spins. The thread chosen is a standard M8, which has a 1.2 mm pitch, which means that one complete rotation of the lever shifts the “Piston” by 1.2 mm. Here, it should be emphasized that the thread in the two parts was chosen to be counterclockwise, as in this way the operation of the device becomes more convenient. This is because it is preferable for the user to turn the lever clockwise to compress the gel with the active substance during operation, whereas moving the lever counterclockwise retracts the plunger. The length of the stroke of the plunger reaches up to 30 mm so that it can cover the entire path to the end of the syringe.

The purpose of this application is to squeeze the cylindrical 3D printed gel with the active substance into the syringe to release the drug. As previously noted, the gel containing the active substance can have any geometry, as it is made with 3D bio-printing. Based on the operating mechanism, the gel is compressed in the syringe, and the active substance that is directed to the microneedles is released. For this reason, the most convenient geometry for this would be a cylinder, which can be easily placed inside the syringe and removed just as easily after use. As the inside diameter of the syringe is 15 mm, the diameter of the gel cylinder is approximately 14 mm.

**Figure 5:**
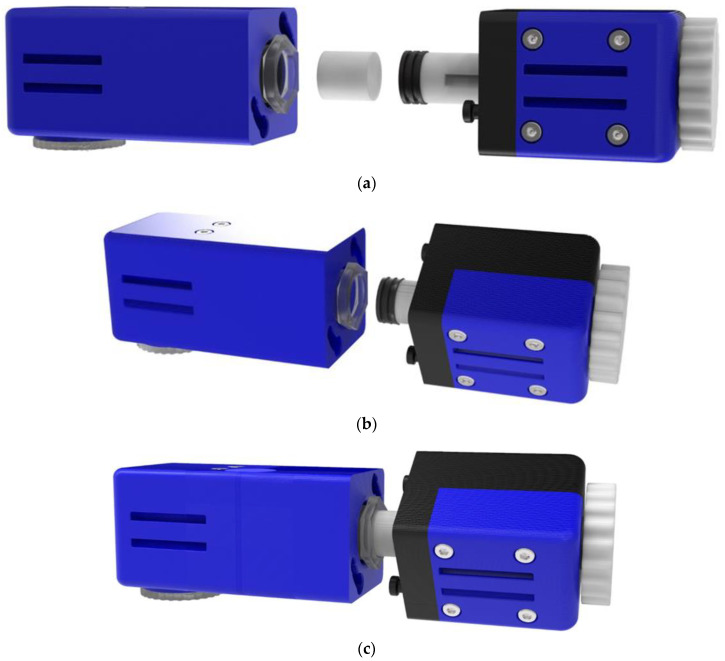

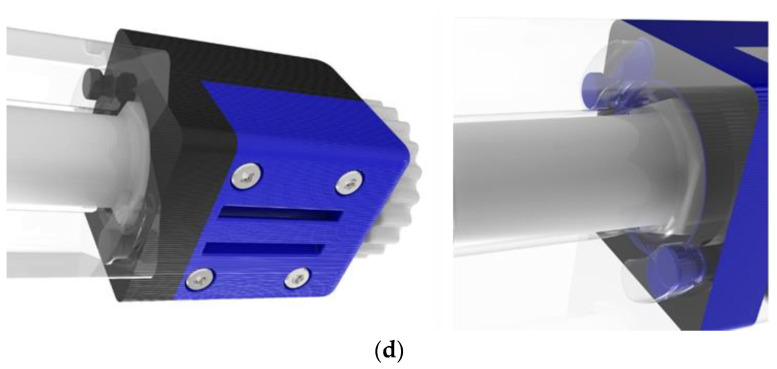
(**a**) Functional illustration of the mechanism and placement of the gel containing the active substance; (**b**) preparation for connection of A and B sub-assemblies; (**c**) pins joining with the cylindrical sockets and coincidence of the surfaces of the two parts; (**d**) rotation and lock of mechanism.

**Figure 6:**
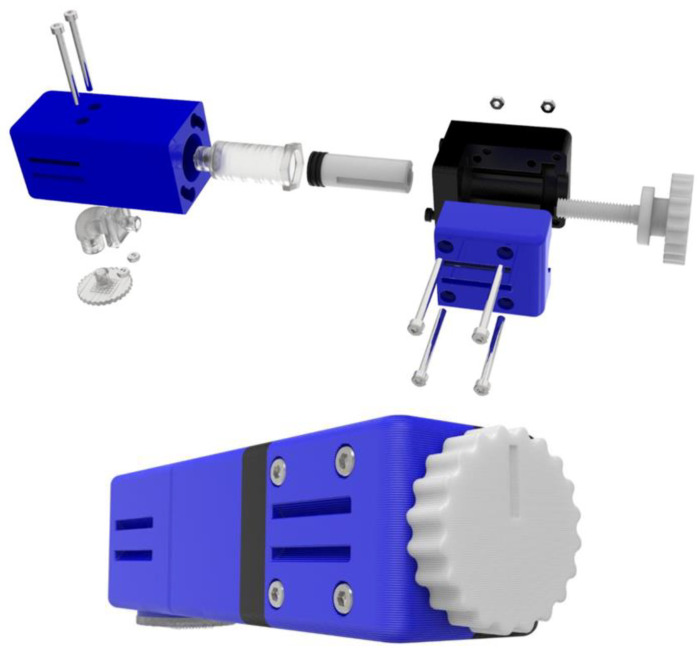
Exploded view of the general assembly of the drug delivery apparatus and as assembled.

The cylinder length is determined by the amount of active substance that should be delivered to the patient’s body. The proposed device allows for using a cylinder that will have a length of up to 30 mm, which will therefore have a total volume of up to approximately 4.5 cm^3^. Regarding the assembly and operation of the application, as illustrated in Figure 5 with photorealistic renders, the cylindrical gel is placed in the syringe and then the union of the two sub-assemblies takes place. To achieve this, we first insert the tip of the “Piston” that has the sealing element into the syringe for a few millimeters to ensure accurate placement. The two sub-assemblies are then connected via specially shaped pins and a female configuration in the “Shell Connection”. Τhe two pins are concentrically inserted into the groove’s large hole. The connection is then secured by rotating sub-assembly B 35° clockwise. To accomplish this, the area where the clasp is made is narrowed just before it is locked. In this way, the levels of the two components and the two layouts coincide. The user then only has to turn the lever clockwise and move the plunger linearly to compress the gel. Thus, the release of the drug from the syringe leads it to the channel that, through the “Angle Connection”, ends up in the microneedles and finally in the subcutaneous tissue of the patient. When this procedure is completed and the device is removed from the patient’s hand, the “MN Array” is first removed, as the gel has the ability, when decompressed, to reabsorb the fluid released during compression. For this reason, liquid absorption should not be allowed again while the microneedles are inserted, as this will transfer germs to the inner parts of the device. After removing the “MN Array” by moving the lever counterclockwise, the piston is moved outwards and then the two sub-assemblies are disconnected in the same way as the union. The gel is then removed with tweezers or another tool. If there is a need to use a new syringe, then the existing syringe can be unscrewed through the hexagon and a new one can be inserted. The control of the rotational movement of the lever enables us to control the amount of medicine that passes to the patient during use. This amount is calculated experimentally based on the speed, controlling the displacement of the plunger along the syringe. To facilitate this process, there is a groove in the “Rotating Lever” so it is known exactly how many spins have been done by hand. Throughout use, the device must be secured to the patient’s hand to ensure tightness. For this reason, there are configurations on the outside to pass a Velcro strap through, as noted above. Figure 6 shows the apparatus in an exploded view as well as in assembled form.

## 3. Fabrication of the Drug Delivery Apparatus

### 3.1. Fused Filament Fabrication Printing

**Figure 7:**
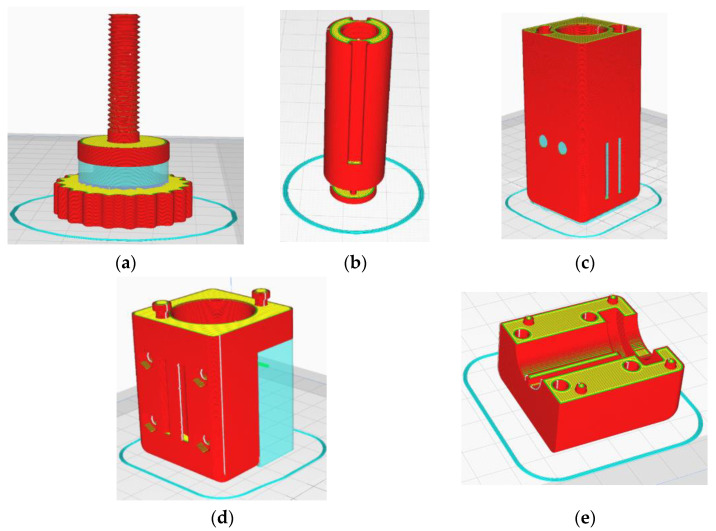
Print simulation in Cura of: (**a**) Rotating Lever; (**b**) Piston with seal; (**c**) Shell; (**d**) Shell Connection; (**e**) Shell Split.

**Figure 8:**
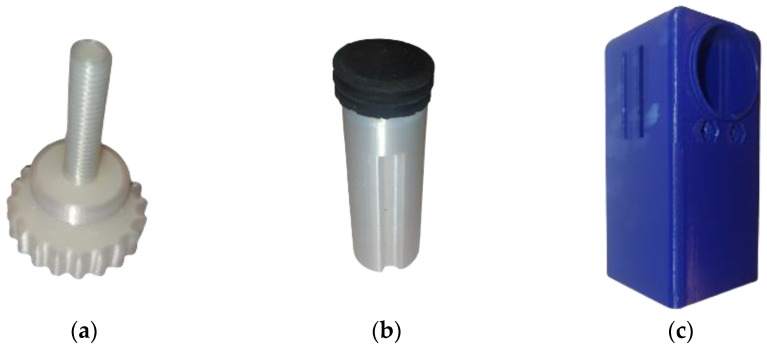

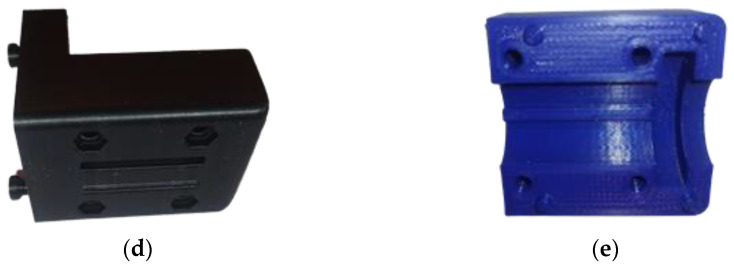
Physical illustration of: (**a**) Rotating Lever; (**b**) Piston with seal; (**c**) Shell; (**d**) Shell Connection; (**e**) Shell Split.

To proceed with the construction of the device described, the choice was based on the most appropriate and cost-effective manufacturing methods. As noted above, the parts should be able to be made with minimal equipment. This fact led to the choice of 3D printing. All the parts were manufactured with the fused filament fabrication (FFF) method, apart from the ‘Angle Connection’, the ‘MN Array, and the ‘Syringe’, which were manufactured with the liquid crystal display (LCD) method in order to have parts with increased accuracy. The BCN 3D sigma FFF 3D printer was used, which has a maximum accuracy of 0.1 mm and can 3D print a 3 mm thick filament. The components of the assembly were 3D printed with a PLA material. All files were designed in Solidworks^ΤΜ^ and exported in STL format before being imported into Cura^TM^ (Figure 7), which was used as a slicer. The 3D printing parameters of the ‘Rotating Lever’ are shown in Table 1 as indicative data, while all 3D printed parts using the FFF technique are illustrated in Figure 8.

The ‘Rotating Lever’ was released in layer thickness 0.1 mm while supports were used in a cylindrical area to print properly. The orientation of the part is such that the length of the thread is printed along the z-axis, which helps to reduce the possible supports but also to produce better thread quality. In addition, the infill density was set to 100% in order to have a solid part, as it receives intense torsional stresses. In this way, it is possible to maximize the robustness of the part, but this increased the printing time to about 4 h.

The ‘Piston’ contains the thread inside and should be connected to the ‘Rotating Level’, which is a key point of the construction. For this reason, a very small layer height was chosen, at 0.1 mm, with an infill density of 80%. The surface standing on the bed was the opposite of the one where the thread starts, to protect its quality as much as possible. Supports were avoided as they are very difficult to remove, and because of this, the seal was difficult to install. The printing time was 1 h and 45 min.

The printing of the ‘Shell’ was made with the large dimension (80 mm) being printed along the z-axis. The upper surface of the part had to be protected, as it plays an important role in the effectiveness of the joint with the ‘Shell Connection’. It is often observed that the surface resting on the 3D printer bed shows roughness, local detachments, and generally more defects. For this reason, the opposite side of the part was chosen to rest on the printer bed, as the surface quality does not play a decisive role in the functionality of the mechanism. Supports were selected everywhere for good rigidity of the many areas of the part that contained notches and grooves. The layer height was 0.2 mm, and the total print duration was about 7 h and 15 min. The infill density was 15%, and the angle was 80°. This led to the complete removal of supports in various places, such as the straps for the strap, but also severely restricted the inside of the workpiece. Regarding the tolerances in addition to the dimensions, this concerned the diameter of the holes for the screws, which became 3.4 mm, whereas the diameter of the housing for the screw head became 5.8 mm. Moreover, an additional margin of 0.3 mm was given on each side of the case inside the part where the ‘Angle Connection’ will nest, as the surfaces cannot be completely smooth, despite the removal of the supports.

Finally, there was a 0.2 mm increase in the stenosis at the point of the clasp. The printing took 6.5 h, and the result was very encouraging, as the parts to which the Shell is connected were easily placed. The ‘Shell Connection’ was printed with 0.16 layer thickness while extensive supports were used to support the upper side. It should be noted here that the part could be printed and rotated 180° so that it does not need any support. However, the functionality of this part is crucial for tying the device through the pins, which should not be put at risk and printed glued to the bed. The infill was selected as 100%, The printing time was 9 h, but we achieved a solid result that did not fail despite repeated tests. The ‘Shell Split’ was placed with the large surface touching the printed bed, so as to avoid any supports. In this way, more accurate printing was achieved on the centering pins but also on the holes through which the screws pass. With layer thickness 0.2 mm and 40% infill density, the printing time was about 3 h and the result was satisfactory.

### 3.2. Semi-Solid Extrusion 3D Printing of the Microgel

**Figure 9:**
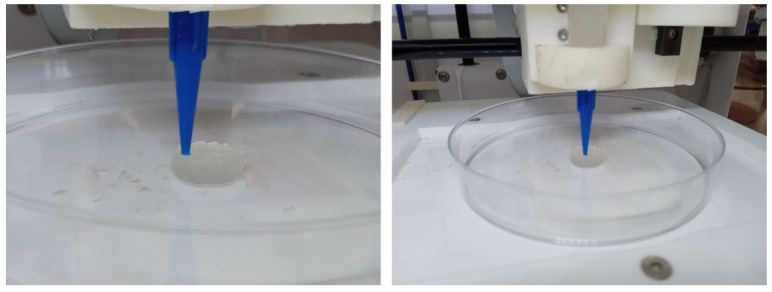
Three-dimensional printing process of the sodium alginate gel cylinders.

**Figure 10:**
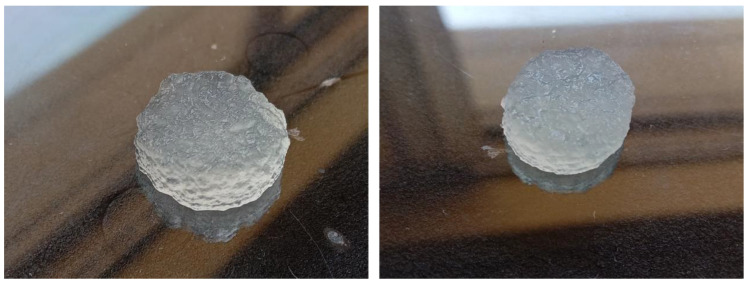
Three-dimensional printed sodium alginate gel cylinders prior to crosslinking.

**Figure 11:**
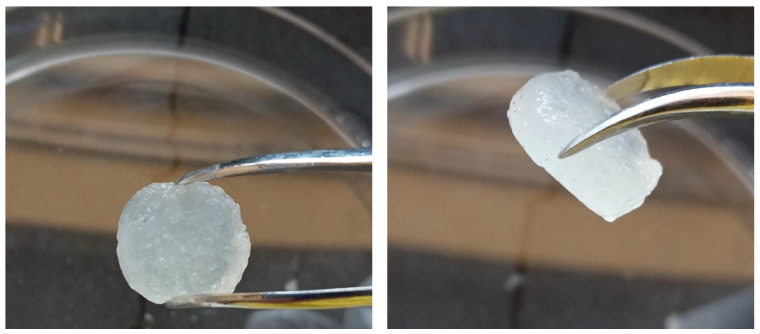
Free-standing 3D printed sodium alginate gel cylinders as the model mechanoresponsive system.

Sodium alginate hydrogels were selected as a model mechanoresponsive [28] material. These hydrogels are composed of a continuous three-dimensional polymeric matrix and an aqueous phase. The mechanoresponsive nature of these gels renders them able to release part of the aqueous phase upon mechanical stimulation without compromising their structure (for low applied external force). To perform in this mechanoresponsive way, these gels should be partially crosslinked to be able to be printed, and subsequently they should be fully crosslinked to become solid. These solid forms are expected to release only part of their aqueous phase (aqueous solutions) suitable for injection. Such a system can be the drug loading carriers. The 3D printing of sodium alginate gels was performed according to the literature, with modification [29,30]. Briefly, 5 mL of a 4% *w*/*v* aqueous solution of sodium alginate (Sigma Aldrich^®^, Merck KGaA, Darmstadt, Germany) was mixed with 0.4 mL of 10% *w*/*v* aqueous solution of calcium chloride (Sigma Aldrich^®^, Merck KGaA, Darmstadt, Germany) to form a viscous printable gel. Cylinders were subsequently printed by an extrusion-based 3D printer (CELLINK^®^ Inkredible, Gothenburg, Sweden). The diameter of the 3D printed semi-solid cylinder can vary accordingly to the dimensions of the microneedle device barrel. The cylinders were submerged by a 10% *w*/*v* aqueous solution of calcium chloride for 5 min to be crosslinked and become free-standing, as illustrated in Figure 9, Figure 10 and Figure 11.

**Figure 12:**
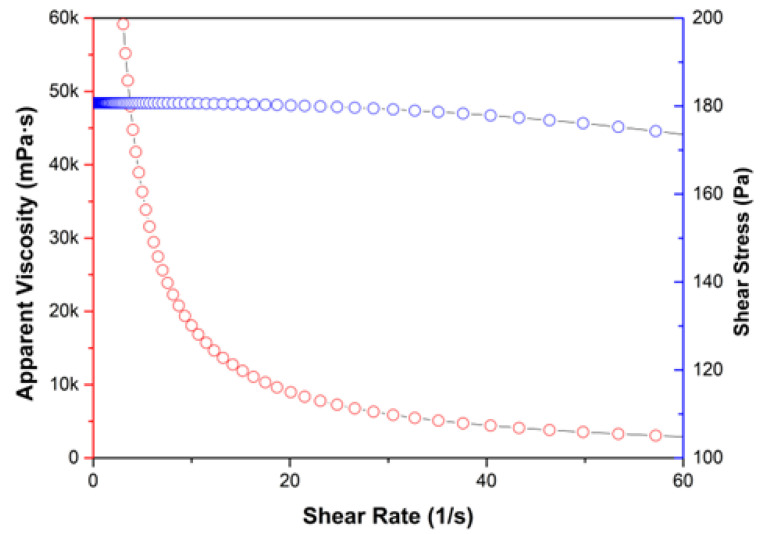
Apparent viscosity and shear stress vs. shear rate plot of the 3D printable sodium alginate gel (3D printing ink).

The rheological behavior of the inks was evaluated using an Anton Paar MCR 92 (Anton Paar GmbH, Graz, Austria) strain rate-controlled rheometer, equipped with a Peltier module. A cone-plate geometry (25 mm diameter) with a 0.5 mm gap was selected. All experiments were performed at 25 °C. The apparent viscosity of the ink formulation, plotted against the shear rate (Figure 12) is a typical example of a shear thinning non-Newtonian liquid, similar to other 3D printable formulations based on alginate in the literature [31,32]. The shear thinning nature of the 3D printable ink is one of the most desirable characteristics of 3D printable ink, as it very important for the formulation to pass easily through the printer nozzle (tip) when it is extruded, subsequently becoming not-flowing after the extrusion.

### 3.3. Liquid Crystal Display Printing

**Figure 13:**
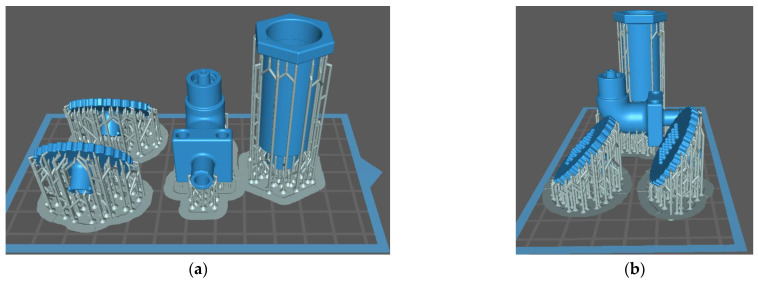

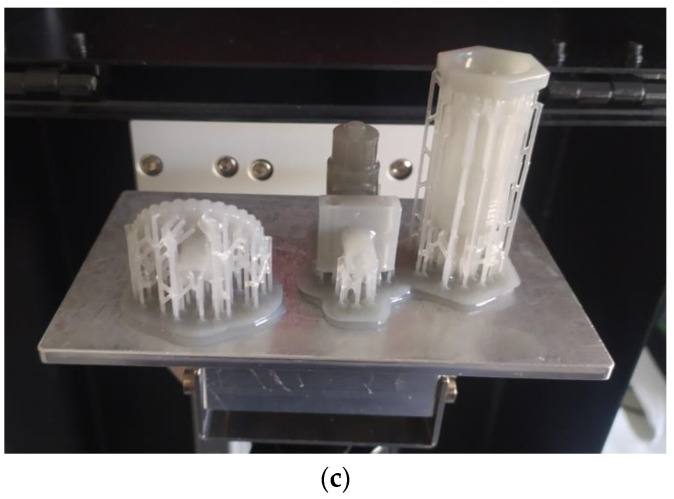
(**a**,**b**) Front and side views of the placement of the parts on the platform with the supports; (**c**) 3D printed components with the platform facing upwards.

Liquid crystal display (LCD) technology was used to build the ‘Angle Connection’, ‘MN Array’, and ‘Syringe’, as illustrated in Figure 13. It is necessary for such parts to have high accuracy and good surface quality, as there are many threads and details that are necessary for the proper assembly and operation of the device. For the formation of hollow microneedles, such a technique is considered especially suitable [20,26]. The design of the parts was achieved using Solidworks^TM^, and then each part was exported to STL separately. The Chitubox^TM^ slicing software was used along with Phrozen Shuffle LCD 3D printer and Phrozen’s matte grey ABS material. Τhe mode of operation of LCD printing involves a build platform that sinks into a resin tank. A digital screen projects the layer throughout the platform, polymerizing all locations at the same time, and such operation continues layer upon layer. For this reason, it is possible to print multiple components in parallel without increasing the printing time, which is determined by the height of the largest part on the z-axis, the layer thickness, and the curing time for each layer. This allows us to print parallel parts at the same time. To adequately adhere the early layers to the construction platform, the space between the tank and the platform was fully cured. Due to the large forces applied on the first layers, longer UV cure times were applied as compared to the subsequent layers. Twelve initial layers were cured with a cure duration of 65 s, whereas 10 s were used for the rest layers. All layer thicknesses were 100 μm, which meets the quality requirements for the printed parts while saving printing time.

Based on the literature, it was decided to print the ‘MN Array’ parts at an angle and with the microneedles on top [20,27]. One component was positioned on the bed at a 45° angle, whereas the second was positioned at 60°. Only light supports with a density of 70% were utilized. Supports are critical components of LCD technique because such structures help prevent deformation and crumbling and are a vital part of the final print. It was decided to print the ‘Angle Connection’ rotated at 180°, as it reduces the need for support. The grid of supports was also increased in density, and several additional ones were placed manually. The ‘Syringe’ was placed with the Luer Lock facing the platform; otherwise, it would need internal supports, which would damage the integrity of the inner surface where the sealing element of the piston penetrates. Three-dimensional printing was executed in 530 layers and lasted a total of 2 h and 14 min, and the total volume of resin used was 18.21 mL, which corresponded to 20 g. The printed items were then placed in isopropyl alcohol using the ultrasonic processor to clean the excess resin. The parts were also cleaned internally by connecting them with a syringe containing isopropyl alcohol so that it flowed through their channels.

**Figure 14:**
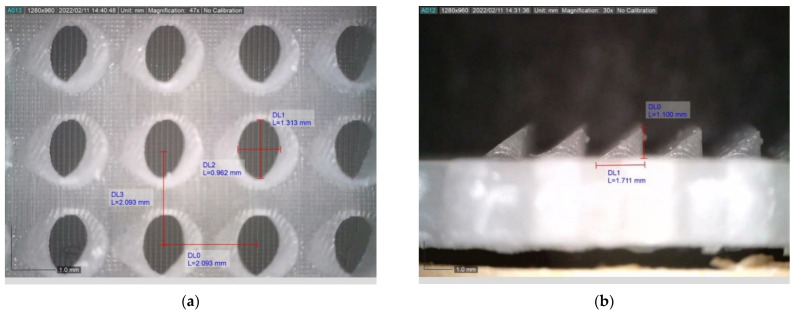
(**a**) Top view and (**b**) side view of the microneedles 3D printed with a liquid crystal display technique.

The 3D printing result of the ‘MN Array’, which required extreme precision, was excellent as far as the geometry of the microneedles is concerned. The microneedles were tested for permeability and the liquid flowed comfortably, without a leak. An optical microscope was also used to capture in detail the geometry of the microneedles, as illustrated in Figure 14. The dimensions of the ‘MN Array’ were measured, such as the distance from center to center of two consecutive microneedles, the height, and the elliptical shape. All measurements were compared with the 3D design and showed very good accuracy. Overall, the construction’s weight was about 200 g, and the external dimensions of the device were 38 × 43 × 140 mm. The ’Syringe’ had a diameter of 15 mm and a length of 30 mm. By utilizing various 3D printing technologies and a simple assembly process, it is possible for non-specialized individuals to manufacture such a cost-effective device, while its ease of use eliminates the need for medical or nursing professionals to be involved in drug administration. The device also enables multiple uses through the removable drug gel and the ‘MN Array’ while it allows control of the amount of drug substance supplied via the rotating lever that converts the rotary motion into a linear one.

**Figure 15:**
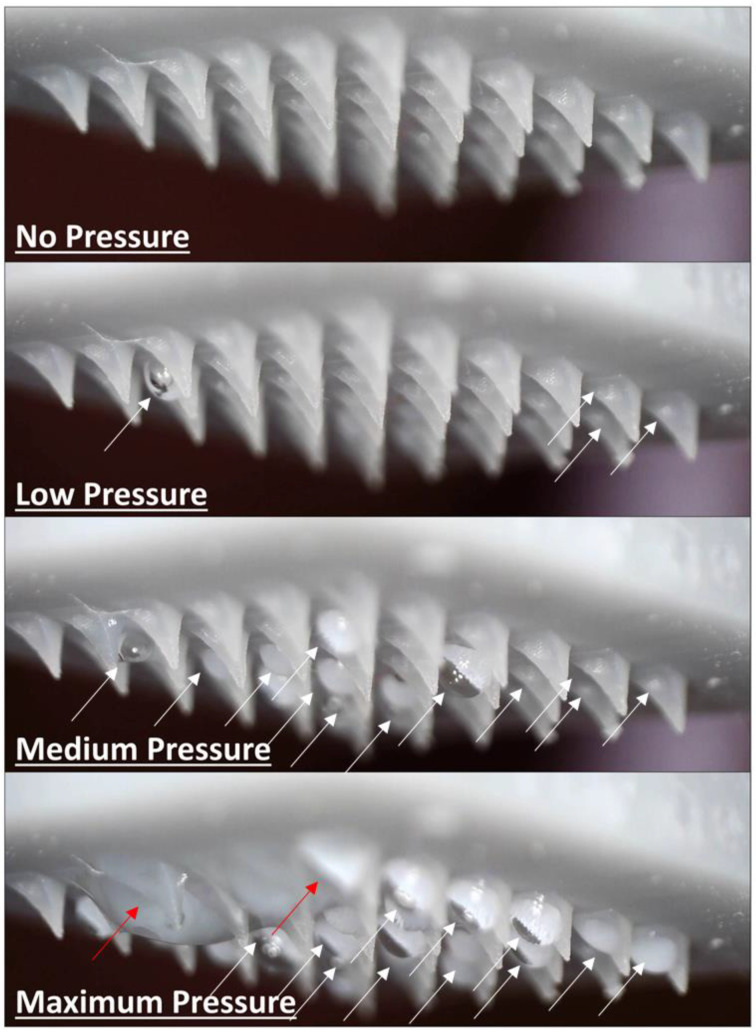
Flow testing of the device at different flow rates. White arrows indicate the distinct liquid droplets formed at individual microneedles, and red arrows indicate coalesced droplets from multiple microneedles that form a continuous superficial liquid film.

Figure 15 shows the injection liquid as it is exerted from the microneedle array at three different pressure levels applied by the device (low, medium, and maximum pressure, respectively). Low pressure is defined as the minimum applied pressure needed for the formation of a distinct droplet by the exerted liquid from the microneedles (white arrows). Maximum pressure, in contrast, is defined as the maximum pressure that can be applied by the device to the crosslinked alginate gel. After the application of the maximum pressure, the gel is fully collapsed and the maximum amount of the injection medium is considered to be released from the 3D printed gel structure. Finally, medium pressure is defined as an intermediate pressure level between low and maximum pressure. Figure 15 shows the gradual formation of distinct droplets during pressure increase (white arrows). At low pressure, the droplets are visible at individual microneedles. At intermediate pressure (medium pressure), droplets are visible at most of the microneedles, whereas at maximum pressure, the liquid droplets coalesce toward the formation of a continuous superficial liquid film (red arrow) that covers multiple microneedles. This behavior is expected as the microneedle array is exposed to air. When the array is inserted in a substrate (skin), there would be no coalescence present in the system during the injection [20].

**Figure 16:**
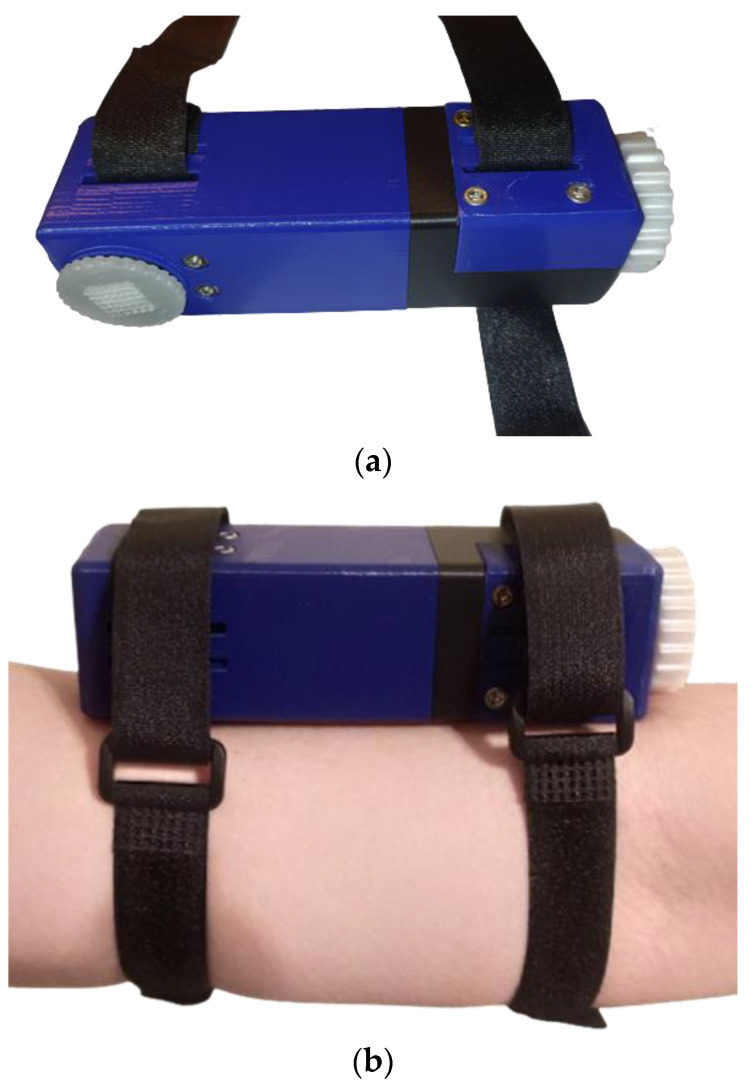
(**a**) Demonstration of the drug delivery apparatus, (**b**) the device attached to the hand of the patient/user using Velcro straps.

To ensure tightness, Velcro straps were utilized to secure the device to the hand of the patient/user, as illustrated in Figure 16. Table 2 describes in detail for each piece of the device (except the microgel), the 3D printing method, the dimensions, the weight, the total layers, and the required printing time.

It should be noted at this point that the main patient risks associated with injection practices, according to the Centers for Disease Control and Prevention, are divided into several categories, and they can be mitigated by following safe injection practices. These practices can be applied to the studied device by taking under consideration the possible similarities and differences. In this context, all the preparations concerning the device should be performed under aseptic conditions or in a clean area. The device is designed in a way that all its components can individually be sterilized, and the materials used can withstand this process. By using mechanoresponsive hydrogels as the drug carrier, the use of vials is avoided; thus, the additional step of disinfecting the rubber septum of the vials can be avoided. Additionally, the solid form of the injection medium renders it easier to handle and store. In any type of injection, the needles or syringes should not be used for more than one patient. In this framework, the device is designed to have disposable parts, such as the needles, the fluidic channel, and the barrel, to avoid accidents. The use of personalized solid sodium alginate mechanoresponsive gels ensures that a single-dose (single-use) medication has been used for no more than one patient, and there is no possible way to combine the leftover contents of single-use vials for later use. Finally, the use of solid sodium alginate mechanoresponsive gels render the microneedle array and fluidic channel not functional after the administration of the dose, in compliance with single-use proofed devices.

## 4. Conclusions

The purpose of this study was to design and construct a microneedle-based drug delivery apparatus. This mechanism achieves controlled drug delivery by adjusting the speed of the external lever in response to the user’s input. The drug-related components feature a standard Luer lock thread and syringe diameter. The prototype was constructed using 3D printing technologies, which are well-suited for such applications as well as conceptual design in general. In the outer parts, the FFF method was used, as it covers our requirements regarding accuracy, while it also has a low cost. For the internal parts, the LCD method was used, as it ensures high precision and surface quality, while enabling the use of biocompatible materials. After fabricating all the parts, the apparatus was assembled using additional components such as screws and a Velcro strap. Finally, the device was evaluated for its ability to transport liquid through the microneedles and for the tightness of the components that enclose the flow channels and their threads. The results established not only the device’s functionality but also its ability to be constructed quickly and easily, which was the device’s initial intent.

Current research efforts of the authors are focused on miniaturizing this by further lowering the device’s volume and hence the amount of material required to create it. Additionally, a computational fluid dynamic (CFD) analysis will be performed in a subsequent phase to determine the pressure loss due to friction along the fluid path. This might be utilized to alter the channel diameters and conserve part of the substance that is lost on the way to the microneedles. In the future, the medication delivery system could be automated, powered by an electric motor, or driven by a linear actuator with accurate piston positioning. This may be directly related to the amount of medication delivered, which the user can select, allowing it to be utilized by anyone without specialized knowledge, so opening up new avenues for drug delivery.

## Figures and Tables

**Table 1:** 3D printing parameters using the FFF method for “Rotating Lever”.

Layer Height (mm)	Print Speed (mm/s)	Flow Rate (mm^3^/s)	Infill Density	Top/Bottom Layers
0.1	50	4.8	15	4
Printing Temp. (°C)	Bed Temp (°C)	Infill Pattern	Support Density	Support Overhang Angle
200	60	Zig-Zag	15%	50^ο^

**Table 2:** Method of printing, external dimensions, weight, total layers of printing, and required printing time for each part of the assembly.

Part Name	Method	Dimensions (W × L × H in mm)	Weight (g)	Total Layers	Time (min)
Rotating Lever	FFF	35 × 49.9 × 35	11.5	500	240
Piston	FFF	14.2 × 42 × 14.2	4	420	105
Shell	FFF	38 × 39.8 × 80	97	400	390
Shell Connection	FFF	38 × 54.7 × 38	32	345	540
Shell Split	FFF	20.8 × 40 × 38	23	104	180
Syringe	LCD	20 × 47.8 × 23	3	530	165
Angle Connection	LCD	18 × 34.5 × 28.9	5.1	340	114
MN Array	LCD	28 × 28 × 11.6	1.6	303	102
